# Sexually Dimorphic Serotonergic Dysfunction in a Mouse Model of Huntington's Disease and Depression

**DOI:** 10.1371/journal.pone.0022133

**Published:** 2011-07-08

**Authors:** Thibault Renoir, Michelle S. Zajac, Xin Du, Terence Y. Pang, Leah Leang, Caroline Chevarin, Laurence Lanfumey, Anthony J. Hannan

**Affiliations:** 1 Howard Florey Institute, Florey Neuroscience Institutes, University of Melbourne, Parkville, Australia; 2 Department of Anatomy and Cell Biology, University of Melbourne, Parkville, Australia; 3 Inserm UMR S894, Paris, France; 4 UPMC, Univ Paris 06, UMR S894, Paris, France; Chiba University Center for Forensic Mental Health, Japan

## Abstract

Depression is the most common psychiatric disorder in Huntington's disease (HD) patients. In the general population, women are more prone to develop depression and such susceptibility might be related to serotonergic dysregulation. There is yet to be a study of sexual dimorphism in the development and presentation of depression in HD patients. We investigated whether 8-week-old male and female R6/1 transgenic HD mice display depressive-like endophenotypes associated with serotonergic impairments. We also studied the behavioral effects of acute treatment with sertraline. We found that only female HD mice exhibited a decreased preference for saccharin as well as impaired emotionality-related behaviors when assessed on the novelty-suppressed feeding test (NSFT) and the forced-swimming test (FST). The exaggerated immobility time displayed by female HD in the FST was reduced by acute administration of sertraline. We also report an increased response to the 5-HT_1A_ receptor agonist 8-OH-DPAT in inducing hypothermia and a decreased 5-HT_2A_ receptor function in HD animals. While tissue levels of serotonin were reduced in both male and female HD mice, we found that serotonin concentration and hydroxylase-2 (TPH2) mRNA levels were higher in the hippocampus of males compared to female animals. Finally, the antidepressant-like effects of sertraline in the FST were blunted in male HD animals. This study reveals sex-specific depressive-related behaviors during an early stage of HD prior to any cognitive and motor deficits. Our data suggest a crucial role for disrupted serotonin signaling in mediating the sexually dimorphic depression-like phenotype in HD mice.

## Introduction

Huntington's disease (HD) is an autosomal dominant neurodegenerative disorder caused by expansion of CAG repeats in exon 1 of the *huntingtin* gene. Clinical onset of HD is determined on the basis of motor symptoms, however the pre-motor stages of the disease are commonly associated with depression [1], [2], [3], [4]

Mood disorders are one of the most prevalent causes of disability worldwide and depression is diagnosed in women at roughly twice the incidence of men in the general population [5], [6]. While HD is an autosomal dominant condition that affects both males and females, there is yet to be a study of sexual dimorphism in the development and presentation of depression in HD patients. However our recent findings in a HD mouse model suggest such clinical investigation is warranted. One hypothesis addressing the sexual dimorphism of depression involves the monoamine neurotransmitter serotonin (5-HT) [7]. Clinical studies suggest gender differences in the associations observed between psychiatric disorders and functional polymorphisms of 5-HT transporter (5-HTT) [8], [9], [10], [11] and tryptophan hydroxylase-2 (TPH2) [12], [13].

The 5-HT_1A_ and 5-HT_2_ receptors are also of particular interest with respect to both pathogenesis and antidepressant efficacy [14]. Major depression has been associated with increased 5-HT_1A_ autoreceptor density in the dorsal raphe nucleus and/or reduced postsynaptic 5-HT_1A_/5-HT_2_ receptor function [15], [16], [17], [18], [19], sometimes in a gender-specific manner [20]. Furthermore, the desensitization of the 5-HT_1A_ autoreceptor has been demonstrated to be essential for enhanced 5-HT transmission following chronic treatment with selective 5-HT reuptake inhibitors [21]. Finally, 5-HT_1A_ and/or 5-HT_2_ receptor abnormalities have been described in different rodent models of mood disorders [22], [23], [24], [25] and HD [26], [27], [28].

The neurobiological basis for the increased incidence of depression in HD remains unclear. Therefore, establishment and validation of an animal model of HD with depression-like behaviors will provide a valuable tool for understanding the mechanisms of the condition as well as the preclinical development of effective therapies. A depressive-like phenotype has recently been observed in two different mouse models of HD [27], [29]. However, these studies did not satisfactorily rule out potential confounds associated with the locomotor/cognitive impairments and did not functionally assess the possible mechanism(s) underlying the depressive-like behaviors.

Using the R6/1 transgenic HD mice which express exon 1 of the mutant human *huntingtin* gene, we recently revealed sex-specific changes in relation to the effects of HD mutation [27], [30]. On the basis of those findings, the current study sought to assess the putative female-specific depression-like behaviors at 8 weeks of age (prior to any cognitive and motor deficits that are observed from 12 and 15 weeks of age respectively in this transgenic line [31], [32]) and further investigate the possible dysregulation of 5-HT signaling in HD animals.

## Results

### Altered affective behaviors in female HD mice

**Figure 1:**
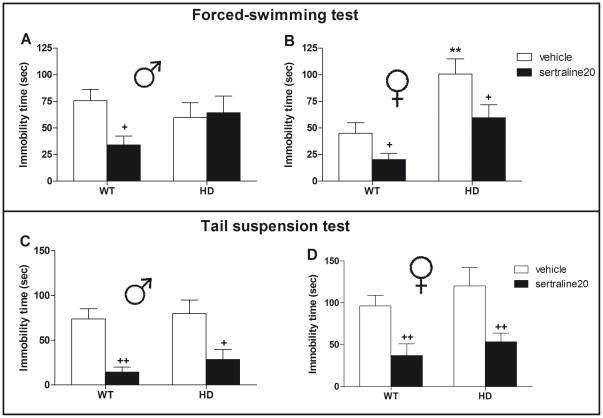
Effect of sex, HD mutation and acute sertraline administration on immobility time in mice exposed to the forced-swimming test (FST) and the tail suspension test (TST). (A) Analyzing FST performances we found no differences in immobility time (in sec) between R6/1 (HD) and wild-type (WT) vehicle-injected male mice. Acute injection with sertraline (20 mg/kg, i.p.) decreased immobility time in male WT mice but not HD animals. (B) Control (vehicle-injected) female HD mice exhibited augmented immobility time when compared to WT animals. The behavioral deficit observed in HD mice was rescued by acute sertraline which also decreased immobility in WT female mice. Looking at immobility time in mice exposed to TST, we found a significant effect of sertraline in both (C) male and (D) female animals without any effect or interaction with the genotype. Values represent means (± SEM) of n = 8–15 mice per group. WT vs. HD: (*) p<0.05; Vehicle vs. sertraline20: (+) p<0.05, (++) p<0.01.

Measuring the time of immobility in the forced-swimming test (FST), we found overall effects of genotype (F_(1,97)_ = 11.7, p<0.001) and treatment (F_(1,97)_ = 10.3, p<0.01) as well as a significant interaction between sex and genotype (F_(1,97)_ = 6.36, p<0.01). We also found a significant interaction between sex, genotype and treatment with sertraline (F_(1,97)_ = 3.85, p<0.05). Indeed compared to WT animals, only female HD mice exhibited augmented immobility times (Fig. 1B). In addition, acute administration of sertraline decreased the mean immobility times of all groups (p<0.05) except for male HD mice (Fig. 1A).

A second cohort of mice was then used to study the effects of acute sertraline on the tail-suspension test (TST) as a second measure of depression-related behavior. Looking at time of immobility, we found overall effects of sex (F_(1,67)_ = 7.35, p<0.05) and treatment (F_(1,67)_ = 33.5, p<0.001) but no effect of genotype (F_(1,67)_ = 2.16, p = 0.15) nor any significant between factors interactions. Indeed regardless of genotype, acute sertraline decreased immobility times of both male (Fig. 1C) and female (Fig. 1D) animals on the TST.

**Figure 2:**
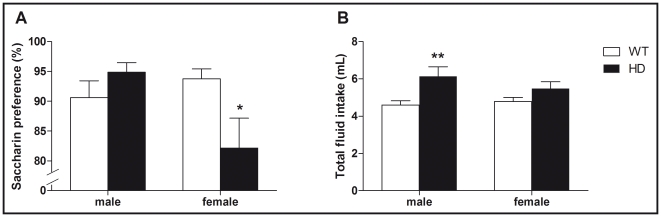
Effect of sex and HD mutation on saccharin-preference test and total fluid intake. (A) We found a significant interaction between sex and genotype (F_(1,44)_ = 5.84, p<0.05) on saccharin preference (expressed as % of total fluid intake). Indeed compared to WT animals, only HD female mice exhibited reduced saccharin preference. (B) Interestingly looking at total fluid intake (expressed in mL), we revealed an overall effect of genotype (F_(1,44)_ = 12.3, p<0.01) but no significant effect of sex or interactions. Values represent means (± SEM) of n = 8–14 mice per group. WT vs. HD: (*) p<0.05, (**) p<0.01.

Anhedonia, defined as a decrease in the perceived pleasant or rewarding properties of events, is a core symptom in depression and can be assessed using the well-validated saccharin preference test [33]. We found a significant interaction between sex and genotype (F_(1,44)_ = 5.84, p<0.05) on saccharin preference (Fig. 2A). Indeed compared to WT animals, only female HD mice exhibited reduced saccharin preference (p<0.05). Interestingly there was an overall effect of genotype (F_(1,44)_ = 12.3, p<0.01) on total fluid intake (Fig. 2B), but no significant effect of sex or interactions.

**Figure 3:**
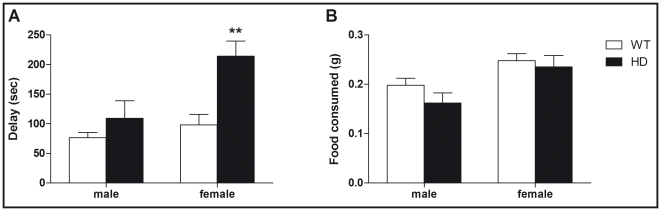
Effect of sex and HD mutation on the novelty suppressed feeding test (NSFT). (A) Analyzing the time (expressed in sec) to complete the task in the NSFT, we found a significant interaction between sex and genotype (F_(1,55)_ = 4.2, p<0.05). Indeed compared to WT animals, only HD female mice exhibited greater delay to complete the NSFT. (B) Interestingly measuring the amount of food consumed after NSFT, we did not find any effect of genotype (F_(1,55)_ = 1.7, p = 0.19) or interaction with the sex (F_(1,55)_ = 0.39, p = 0.54). Values represent means (± SEM) of n = 12–17 mice per group. WT vs. HD: (***) p<0.001.

In order to extend the study of the emotionality-related behaviors, mice were exposed to the novelty-suppressed feeding test (NSFT) in which we measured the latency of the animal to eat in an aversive environment. This paradigm provides an anxiety-related measure and has been shown to be sensitive to both anxiolytics and chronic antidepressants [34]. Analyzing the latency to feed (Fig. 3A), we found a significant effect of both sex (F_(1,55)_ = 9.6, p<0.01) and genotype (F_(1,55)_ = 13.3, p<0.001) as well as a significant interaction (F_(1,55)_ = 4.2, p<0.05). Since latency data failed to pass the normality test (p<0.05, Shapiro-Wilk test), we also ran non-parametric tests and found an overall significant group difference (p<0.01, Kruskal Wallis test). Compared to WT animals, only female HD mice exhibited augmented time to feed (p<0.01, Mann-Whitney test). Our observations were not due to altered appetite motivations since there was no difference in the amount of food consumed after NSFT (Fig. 3B).

### Sex-specific increased 8-OH-DPAT-induced hypothermia in female HD mice

**Figure 4:**
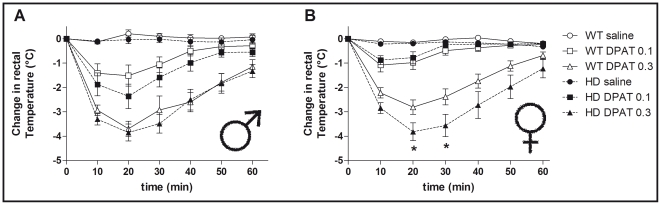
Effect of sex and HD mutation on 8-OH-DPAT-induced hypothermia. Acute injection with the 5-HT_1A_ receptor agonist 8-OH-DPAT (0.1 and 0.3 mg/kg, s.c.), decreased rectal temperatures in a dose-dependent manner in both (A) male and (B) female mice when compared to saline-treated animals. However, only female HD mice exhibited an augmented response to the high dose of 8-OH-DPAT (0.3 mg/kg). The maximum hypothermic response to 8-OH-DPAT was observed 20 min post-injection in both WT/HD male (−3.69±0.31 and −3.85±0.35°C respectively) and WT/HD female (−2.81±0.30 and −3.88±0.36°C respectively, p<0.05). Values are expressed as change of temperature (°C) compared to baseline and represent means (± SEM) of n = 8–14 mice per group. WT vs. HD: (*) p<0.05.

We assessed the hypothermic response to the 5-HT_1A_ receptor agonist 8-OH-DPAT to evaluate 5-HT_1A_ autoreceptor function *in vivo*. The involvement of 5-HT_1A_ autoreceptors in mediating the 8-OH-DPAT-induced hypothermia is well-established in the mouse [35] but also see [36], [37]. There were no differences in baseline temperatures between the sexes or genotypes prior to drug administration (data not shown). Considering the change in body temperature for 1 h post-injection (Figure 4), we found that acute administration of 8-OH-DPAT significantly decreased rectal temperatures in a dose-dependent manner in both male (Fig. 4A) and female (Fig. 4B) mice compared to saline-treated animals (treatment effect: F_(2,116)_ = 142, p<0.001). There were significant effects of both sex (F_(1,116)_ = 6.48, p<0.05) and genotype (F_(1,116)_ = 4.61, p<0.05) as well as significant interactions between the 3 factors. Indeed 20–30 min following 8-OH-DPAT (0.3 mg/kg) administration, only female HD mice exhibited an enhanced hypothermia when compared to WT animals (p<0.05).

### Functional analysis of 5-HT_1A_ and 5-HT_2_ heteroreceptors

**Figure 5:**
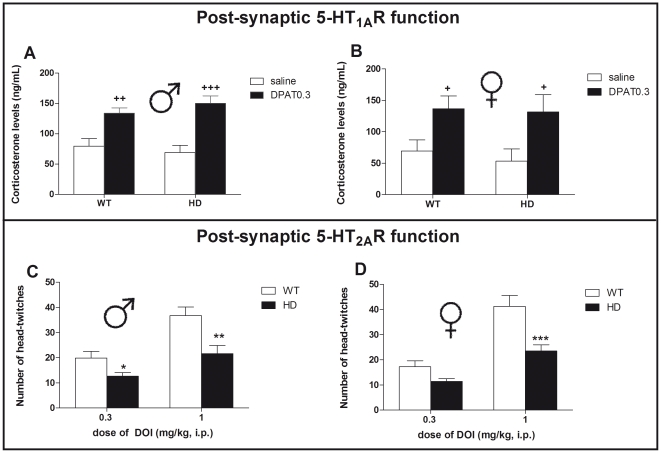
In vivo assessment of 5-HT_1A_ and 5-HT_2_ post-synaptic receptor function using 8-OH-DPAT-induced change of corticosterone levels and DOI-induced head-twitches. Compared to paired-saline injected animals, administration of the 5-HT_1A_ agonist 8-OH-DPAT (0.3 mg/kg, s.c.) significantly increased corticosterone levels in both (A) male and (B) female mice, regardless of the genotype. In the DOI-induced head-twitches experiment (bottom panel), there was a significant effect of genotype in both sexes. Indeed, the number of head-twitches following DOI (1 mg/kg) were decreased in (C) HD male and (D) HD female mice when compared to WT animals. Values represent means (± SEM) of n = 5–10 mice per group. Saline vs. DPAT0.3: (+) p<0.05, (++) p<0.01, (+++) p<0.001 WT vs. HD: (*) p<0.05, (**) p<0.01, (***) p<0.001.

We assessed 5-HT_1A_ post-synaptic receptor function in vivo, by measuring the effect of the 5-HT_1A_ receptor agonist 8-OH-DPAT on the plasma corticosterone levels in both male and female animals. Regardless of sex and genotype, the acute administration of 8-OH-DPAT (0.3 mg/kg, s.c.) significantly increased corticosterone levels (Fig. 5A & 5B). There was a significant overall drug effect (F_(1,38)_ = 31.7, p<0.001) but no effect of sex or genotype.

The administration of the 5-HT_2A/2C_ receptor agonist (±)DOI, dose-dependently induced head-twitches that were reduced in both male (Fig. 5C) and female (Fig. 5D) HD mice compared to their respective WT control groups. There were significant effects of genotype (F_(1,61)_ = 32.8, p<0.001) and treatment (F_(1,61)_ = 59.8, p<0.001), but no effect of sex (F_(1,61)_ = 0.99, p = 0.75).

### Effect of the HD mutation on gene expression in the raphe

**Figure 6:**
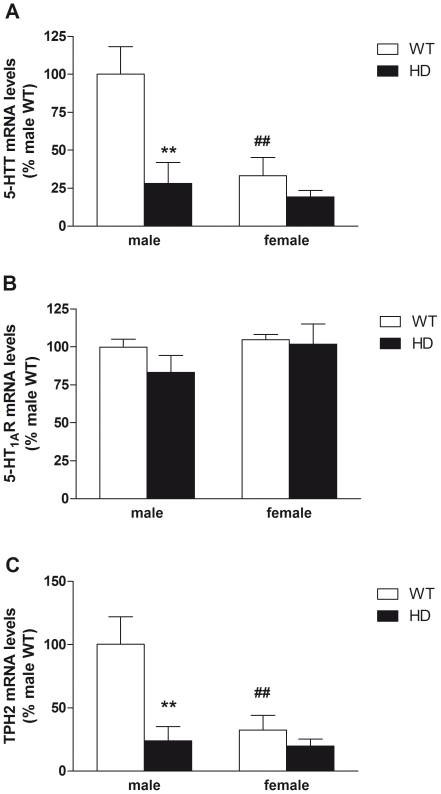
Gene expression of 5-HT transporter (5-HTT), 5-HT_1A_ receptor (5-HT_1A_R) and tryptophan hydroxylase-2 (TPH2) in the raphe. Measuring mRNA levels of several genes encoding proteins that regulate 5-HT homeostasis, we found significant genotype-sex interactions for both mRNA levels of (A) 5-HTT and (B) TPH2 but not for (C) 5-HT_1A_R gene expression. Both 5-HTT and TPH2 mRNA levels were decreased in female WT when compared to male WT. In addition, HD mutation decreased both 5-HTT and TPH2 gene expressions in male only. Finally 5-HT_1A_R mRNA levels were not affected by either the sex or the genotype. Values represent means (± SEM) of n = 5–6 mice per group. WT vs. HD: (**) p<0.01. Male vs. female: (##) p<0.01.

Analyzing mRNA levels of several targets involved in serotonin homeostasis (Figure 6), we found an overall significant effect of sex and genotype for both 5-HTT (F_(1,19)_ = 8.9, p<0.01 and F_(1,19)_ = 10.4, p<0.01 respectively) and TPH2 (F_(1,19)_ = 6.45, p<0.05 and F_(1,19)_ = 9.9, p<0.01 respectively). Interestingly, there were genotype-sex interactions for 5-HTT (F_(1,19)_ = 4.8, p<0.05) and TPH2 (F_(1,19)_ = 5.1, p<0.05). Post-hoc analysis showed that both 5-HTT and TPH2 mRNA levels were decreased in female WT when compared to male WT (p<0.01). In addition, HD mutation decreased both 5-HTT and TPH2 gene expression in males only (p<0.01). In contrast, 5-HT_1A_ receptor mRNA levels (Fig. 6B) were not affected by either sex (F_(1,19)_ = 1.5, p>0.05) or genotype (F_(1,19)_ = 1.0, p>0.05).

### Tissue levels of serotonin and its metabolite in HD mice

**Figure 7:**
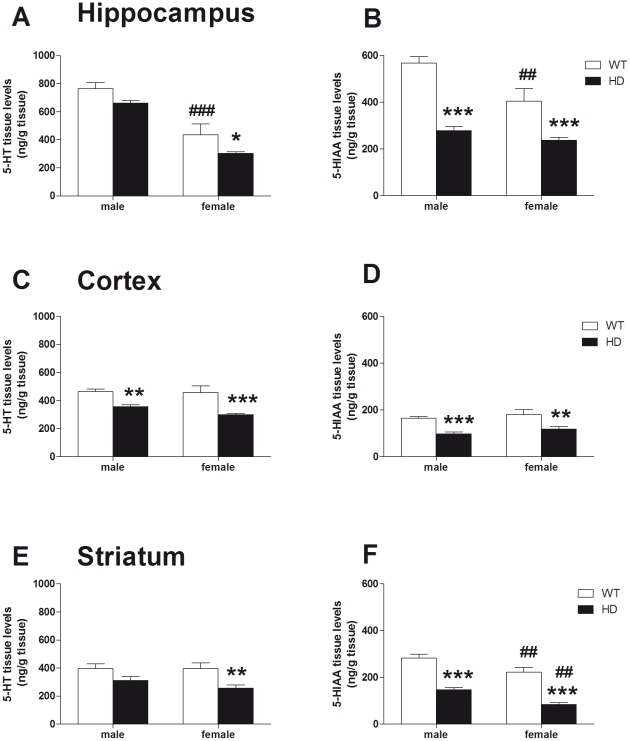
Concentrations of 5-HT and 5-HIAA in brain tissue. Using the HPLC system, we measured the tissue levels of serotonin (5-HT) and its metabolite 5-hydroxyindolacetic acid (5-HIAA) in several brain areas. Both 5-HT and 5-HIAA concentrations were decreased in the hippocampus (A/B), the cortex (C/D) and the striatum (E/F) in female HD mice when compared to WT animals. Levels of 5-HIAA were also reduced in male HD. Finally, male mice exhibited higher hippocampal tissue levels of both 5-HT and 5-HIAA when compared to female animals. 5-HT and 5-HIAA are expressed in ng/g tissue. Values represent means (± SEM) of n = 6–8 mice per group. WT vs. HD: (*) p<0.05, (**) p<0.01, (***) p<0.001. Male vs. female: (##) p<0.01.

In order to assess the effect of the HD mutation on the global serotonergic activity, we measured the levels of serotonin (5-HT) and its metabolite (5-HIAA) in several brain areas (Figure 7) including hippocampus (panels A&B), cortex (panels C&D) and striatum (panels E&F). In each of the three brain regions studied, we found an overall effect of genotype for both 5-HT and 5-HIAA tissue levels. Post-hoc tests revealed significant reductions of 5-HT and/or 5-HIAA levels in HD tissue compared to WT mice in both sexes. We also found a significant effect of sex analyzing both 5-HT and 5-HIAA levels in hippocampus (F_(1,23)_ = 65.6, p<0.001 and F_(1,23)_ = 13.2, p<0.001, respectively) as well as striatal 5-HIAA levels (F_(1,23)_ = 19.3, p<0.001). Indeed, in these two regions female WT mice exhibited decreased 5-HIAA levels when compared to control male animals. Interestingly, the 2-way ANOVA revealed significant interaction between sex and genotype only for hippocampal 5-HIAA levels (F_(1,23)_ = 4.58, p<0.05).

## Discussion

Despite evidence of affective disorders in HD and the known susceptibility of female to developing depression in the general population, there has yet to be a study of sexual dimorphism in the development and presentation of depression in HD patients.

We report here a sexually dimorphic depression-like phenotype in the R6/1 HD mice at 8 weeks of age, prior to the development of any motor and/or cognitive deficits which may have confounded previous studies [27], [29]. We found that latency to eat food in the NSFT as well as immobility times in FST were both specifically prolonged in female but not in male HD mice, and that the acute administration of the SSRI sertraline corrected this latter behavioral impairment. Indeed in the FST, acute injection with sertraline significantly decreased the immobility time of HD animals (when compared to the HD vehicle-injected group), leading to similar FST performances as those displayed by WT controls (vehicle-injected group). In contrast, the HD mutation did not affect TST performance at 8 weeks of age, consistent with the view that distinct neurochemical pathways mediate responses to FST versus TST [27], [38], [39]. These TST results are in contrast with a previous study [27] which reported a conflicted and unexpected “anti-depressive” phenotype displayed by female HD mice exposed to TST at 12 weeks of age, and support the relevance of studying depression-like behaviors in HD at an earlier stage of the disease. As an indicator of altered reward system function, the female but not male HD mice displayed a decreased preference for saccharin. This is the first report of such sex-specific behavioral deficits in R6/1 HD mice, since Pang et al. (2009) focused on behavioral tests modeling the experience of helplessness. Because the diagnosis of a depressive episode requires that an individual has chronically experienced at least 2 out of the 3 core symptoms (according to the ICD-10 criteria: low mood, fatigue and anhedonia), it is critical to used a broad battery of behavioral tests to satisfactorily characterize a depressive-like phenotype in mice. Collectively, our present findings support the well-established gender differences in the vulnerability to depression reported in general population [5], [6]. Similar results have been found in other animal models of depression [40], [41] and female-specific impairments have already been reported using other rodent models of HD [42], [43], [44].

Sertraline decreased FST/TST immobility times of female HD mice to the same extent as WT controls. A similar antidepressant effect seemed to occur in male animals exposed to TST in which both genotypes responded to the drug. In contrast, but consistent with previous findings at 12 weeks of age [27], acute sertraline elicited an antidepressant-like effect in WT but not HD male mice in FST, once again suggesting distinct pathways for response to FST versus TST. Sex-differences in response to sertraline have been described in chronic depression, with women being more likely to show a favorable response to SSRIs compared to men [45]. Several targets have been proposed in mediating the antidepressant-like effect of acute sertraline [46], [47], [48] and it would be too simplistic to draw conclusions based on the correlation between immobility times in FST and 5-HTT mRNA levels within a unique brain region. Indeed, we found that WT females had lower 5-HTT mRNA levels than WT males in the raphe but still responded to acute sertraline (however we previously reported no sex differences for hippocampal and cortical 5-HTT expression, [27]). All together our findings suggest that lower 5-HTT gene expression may be associated with vulnerability to depression in female mice but could have opposite effects on male animals. Similarly women and men carrying the less transcriptionally efficient 5-HTTLPR short allele display opposite responses to environmental stress factors, suggesting that the molecular mechanisms underlying depression may differ between sexes [8], [10]–[11].

Another present finding which helps explain the female-specific depression-related behaviors, is the enhanced hypothermic response to the 5-HT_1A_ receptor agonist 8-OH-DPAT in female but not in male HD mice. The desensitization of the 5-HT_1A_ autoreceptor localized in the dorsal raphe nucleus (DRN) is essential for enhancement of 5-HT transmission following chronic SSRI treatment [21], [49] and sex differences in the adaptive regulation of the 5-HT_1A_ receptor-mediated responses have been previously observed in mice [50], [51], [52]. In mice, 8-OH-DPAT-induced hypothermia seems to be mediated by somato-dendritic 5-HT_1A_ autoreceptors [35]. However some data suggest that the model may be more complex [36], [37] and that other receptor(s) and/or region(s) might be involved. In that regard change in mRNA levels was not consistent with the increased response to 5-HT_1A_ agonist we observed in female HD mice, however 5-HT_1A_ receptor protein or binding was not examined in this present study and may not have necessarily changed in the same direction as the mRNA levels. Additional experiments (e.g. GTPgammaS or electrophysiology) would be required to assess the 5-HT_1A_ autoreceptor function specifically in the raphe.

Collectively, our results suggest that the female-specific depression-like behavior was associated with an increased response to 8-OH-DPAT and a global reduction of serotonergic transmission as suggested by the decreased tissue levels of serotonin (5-HT) and its metabolite (5-HIAA) in various brain areas of HD animals. The 5-HT and 5-HIAA deficits observed in our 8-week-old female R6/1 HD mice are consistent with similar findings in the R6/2 model [53]. Interestingly in male HD mice, 5-HT concentration was reduced only in the cortex although levels of 5-HIAA were massively decreased in the three regions studied. These observations suggested an impaired serotonin turnover in male HD. Detailed exploration of the enzymes involved in 5-HT catabolism (e.g. monoamine oxidase, aldehyde dehydrogenease) would be informative to address this matter. In addition, both hippocampal serotonin content and TPH2 mRNA levels were higher in male when compared to female mice. Whether such sex differences in neurochemistry are the cause or the consequence of the reported sex-dependent 5-HT_1A_ autoreceptor dysregulation, is an important point to address in the future to facilitate understanding of the pathophysiology of depression.

Supersensitivity of 5-HT_1A_ autoreceptors has already been reported in other animal models of depression [22], [24], [54] but also see ([55], [56]). In addition, enhanced 5-HT_1A_ autoreceptor function has been associated with reduced serotonergic neurotransmission (as a lower baseline firing rate of DRN 5-HT neurons) in several models of depression [39], [57] and a significant negative correlation between serotonin synthesis and 5-HT_1A_ binding potential has been reported in healthy volunteers [58]. Interestingly, an increased 5-HT_1A_ receptor density has also been observed in the DRN of depressed patients [18], [59]. However, there exists disagreement within the literature regarding the presence and direction of 5-HT_1A_ receptor abnormalities [15], [60]. Interestingly, a sensitization of 5-HT_7_ receptors in HD mice cannot be excluded since the involvement of these receptors in 8-OH-DPAT-induced hypothermia has been reported in mice [37]. In addition, selective 5-HT_7_ receptor antagonists have been recently proposed as a new class of antidepressants [61]. Whether such an adaptive change occurs in HD mice is an interesting question to be addressed in future studies.

The brain serotonin deficits reported in our 8-week-old female HD mice are more likely to be driven by the resultant increased 5-HT_1A_ receptor-mediated inhibitory feedback on serotonergic neuronal firing than impairment in the synthesis process. Indeed in females, we did not find any genotype effect on the gene expression of TPH2, the rate-limiting enzyme in the biosynthesis of 5-HT. However, this interpretation is limited by a possible floor effect in female mice (which showed lower levels of mRNA relative to males). In contrast, TPH2 mRNA levels were reduced in HD male mice when compared with control animals, although 5-HT concentrations were not affected by the mutation, at least in male hippocampus and striatum. Male HD mice could possibly have developed adaptive changes to counteract the TPH2 deficit. Whether such adaptation(s) occur in a region-specific manner would be an interesting question to address. Interestingly, recent data suggest that differences in brain 5-HT synthesis may be larger between healthy and depressed men that between healthy and depressed women [62]. Consistent with our observations in WT animals, it has been found that females had lower levels of mRNA relative to males quantifying mRNA in post-mortem human brain [63]. In addition, the mean rate of serotonin synthesis in healthy males was found to be 50% higher than in normal females [64].

Interestingly, a sex-specific increased in Hypothalamic-pituitary-adrenal (HPA) axis reactivity has been recently reported in women with chronic major depressive disorder [65]. As well as an increased levels of the stress hormone (corticosterone/cortisol) in both R6/2 HD mice and late-stage HD patients [66]. We therefore assessed the effect of 5-HT_1A_ post-synaptic receptor activation on corticosterone levels [52] in both male and female HD mice. The similar 8-OH-DPAT-induced increase in corticosterone in both WT and HD animals was unexpected given that HD mice have been demonstrated to have decreased 5-HT_1A_ receptor density [28] and gene expression [27] in the hippocampus and the cortex. However, it is possible that the hypothalamic 5-HT_1A_ receptors (involved in mediating corticosterone release) were not affected by the HD mutation or underwent additional opposite adaptive changes. This putative adaptation could be related to the altered DPAT-induced hypothermia and the increased fluid intake (previously observed in R6/2 HD mice [67]) we reported here. Indeed, the hypothalamus is known to be involved in the regulation of body temperature and water balance. Whether such putative region-specific changes are link to the hypothalamic capacity to regulated endocrine activity still need to be addressed.

We also report for the first time a reduced 5-HT_2_ receptor function in HD animals. These data demonstrate that the HD mutation disrupts 5-HT_2_ receptor responses, and could be associated with previously described decreases of 5-HT_2A/2C_ receptors in models of HD [26], [27]. However, even though such alterations have been observed in other mouse models of depression [68], [69], the reduced 5-HT_2_ receptor function was displayed by both male and female HD mice and could not explain *per se* the female-specific depression-like behaviors. Interestingly, variation in levels of 5-HTT expression has been shown to be a source of changes in 5-HT_2A_ receptor function [70].

In conclusion, we have found that the female-specific depression phenotype of the R6/1 model is associated with decreased levels of serotonin and its metabolite across various brain regions, which are likely due to altered 5-HT_1A_ receptor signaling as suggested by the increased 8-OH-DPAT-induced hypothermia displayed only by female HD mice. The identification of this specific molecular abnormality presents as a novel target for the early treatment or prevention of depression in HD. Finally even though male HD animals did not display any impaired emotionality-related behaviors, they shared some aspects of molecular dysfunction with the female HD mice such as a decreased 5-HT_2A_ receptor function when compared to WT animals.

## Materials and Methods

### Ethics Statement

All experiments were performed in accordance with the guidelines of the HFI Animal Ethics Committee (approval ID, AEC #08-100) and the National Health and Medical Research Council (NHMRC).

### Animals

R6/1 transgenic hemizygote males [71] were originally obtained from the Jackson Laboratory (Bar Harbor, ME, USA) and bred with CBB6 (CBA×C57/B6) F1 females to establish the R6/1 colony at the Howard Florey Institute (HFI). After weaning, animals were grouped housed (4 mice per cage with 2 of each genotype) and maintained on a 12 h light/dark cycle with access to food and water *ad libitum*. To avoid any possible “litter effects”, we ensure that animals from each litter were represented in each experimental condition using an appropriate randomization. All experiments were performed on male and female wild-type (WT) and R6/1 (HD) mice at 8 weeks of age and in accordance with the guidelines of the HFI Animal Ethics Committee (approval ID, AEC #08-100) and the National Health and Medical Research Council (NHMRC). Each animal was only exposed to a unique behavioral test.

### Drugs

The 5-HT_2A/C_ receptor agonist, 1-(2, 5-dimethoxy-4-odophenyl)- 2-aminopropane ((±)DOI) and the 5-HT_1A_ receptor agonist, 8-hydroxy-di-n-propylaminotetralin ((±)-8-OH-DPAT) were supplied by Sigma (Aldrich, NSW, Australia).

### Body Temperature

Core temperature was measured at ambient temperature of 23±1°C in gently restrained mice using a thermocouple probe (ID-Tech-Bioseb, France; 0.71 mm diameter).

### Forced-Swimming Test (FST)

Mice were individually placed into a glass beaker (13 cm diameter) filled with 12 cm deep water (25–26°C) and video recorded for 300 secs. Total immobility time was manually scored by an experienced experimenter blind to treatment and mouse genotype. To study the effects of acute sertraline (Pfizer Inc, CT, USA), mice were injected intraperitoneally (i.p.) with either vehicle (1% Tween-20, 1 ml/100 g body weight) or sertraline (20 mg/kg) 60 mins before FST [27]. A total of 105 animals (48 males and 57 females) were assessed on FST (n = 10–15 mice per group).

### Tail-Suspension Test (TST)

Mice were suspended by the tail to a hook using adhesive tape. The duration of immobility over a 6-min session was manually scored by an experienced experimenter blind to treatment and mouse genotype. To study the effects of acute sertraline (Pfizer Inc, CT, USA), mice were injected intraperitoneally (i.p.) with either vehicle (1% Tween-20, 1 ml/100 g body weight) or sertraline (20 mg/kg) 30 mins before TST [27]. A total of 75 animals (41 males and 34 females) were assessed on TST (n = 8–12 mice per group).

### Novelty-Suppressed Feeding Test (NSFT)

As previously described [27], mice were food deprived for 48-hours prior to testing but allowed 2-hours of feeding after an initial 24-hour period. Water was available *ad libitum*. During the test, a single food pellet was placed on a piece of filter paper in the centre of the test arena (80×80×80 cm). Individual mice were then placed into a corner of the arena and the latency to grasp and feed on the food pellet within 5 minute was recorded. Upon the initiation of feeding or reaching the time limit, mice were removed and allowed to feed on a single food pellet of pre-determined weight for 5 minutes (to assess the level of hunger). A total of 59 animals (29 males and 30 females) were assessed on NSFT (n = 12–17 mice per group).

### Saccharin-preference test

Based on a validated protocol [33], single housed mice were exposed to both 0.1% saccharin and tap water solutions across four 15 h-overnight periods (18:00 h to 9:00 h). To avoid place preference, the position of the 2 bottles was varied every day across the left or right side of the feeding compartment. In order to take into account time for the animals to become accustomed to the testing environment, saccharin preference score (saccharin intake/total fluid intake) was calculated as the mean of the 2 last measures. A total of 48 animals (22 males and 26 females) were assessed on saccharin preference test (n = 8–14 mice per group).

### 8-OH-DPAT-induced hypothermia

As previously described [39], basal values were determined just before subcutaneous injection of the 5-HT_1A_ receptor agonist 8-OH-DPAT (0.1 and 0.3 mg/kg) or vehicle (0.9% NaCl, 1 ml/100 g body weight), and body temperature was measured every 10 min thereafter. The response to 8-OH-DPAT was calculated as the decrease (from baseline) in body temperature during 60-min post injection. A total of 128 animals (58 males and 70 females) were used for 8-OH-DPAT-induced hypothermia (n = 8–14 mice per group).

### 8-OH-DPAT-induced change of serum corticosterone levels

Mice naïve to behavioral testing were killed 60-min after acute subcutaneous injection of the 5-HT_1A_ receptor agonist 8-OH-DPAT (0.3 mg/kg) or vehicle, between 8.30–10.00am. Blood was collected, left to clot at room temperature for 30 mins and centrifuged at 1000 g for 15 mins. Serum was then collected and corticosterone quantified with an Enzyme Immuno Assay (Cayman Chemical Company, Ann Arbor, MI, USA). A total of 46 animals (23 males and 23 females) were used for 8-OH-DPAT-induced corticosterone release (n = 5–7 mice per group).

### DOI-induced Head-Twitches

Mice were administered with vehicle or the 5-HT_2A/C_ receptor agonist (±)DOI (0.3 and 1 mg/kg, i.p.) and immediately placed inside an observation area. The number of head twitches was counted for a 15-min period starting 15 min after drug administration. A total of 69 animals (32 males and 37 females) were used for DOI-induced head-twitches (n = 8–10 mice per group).

### Real-time PCR for quantification of mRNA expression

Mice naïve to behavioral testing were killed for dissection of raphe. Total RNA was isolated using Qiagen RNeasy extraction kits (Qiagen, NSW, Australia) and stored at −80°C. RNA concentration and quality were determined using Nanodrop spectrophotometer. cDNA was reverse transcribed from 1 mg of total RNA per sample using Applied Biosciences Reverse Transcription kits (PE Applied Biosystems, Foster City, CA, USA) with random hexamers. The reverse transcription PCR conditions were 25°C—10 min, 48°C—30 min and 95°C— 5 min. cDNA products were stored at −20°C. Quantitative real-time PCR was performed using SYBR Green PCR master mix (Sigma-Aldrich) on a PE ABI Prism 7500 Light Cycler system (PE Applied Biosystems). All primer pairs were optimized for working concentrations before use and primer sequences are as follows. Serotonin 1A receptor (5-HT_1A_R) F: CCC CAA CGA GTG CAC CAT, R: GCG CCG AAA GTG GAG TAG AT; Serotonin transporter (5-HTT) F: CTT CAG CCC CGG ATG GTT, R: GTG GAC TCA TCA AAA AAC TGC AAA; TPH2 F: GAC AAC GTG CCG CAA CTG R: TGA AGC CAG ATC GCT CTT TCA; Cyclophilin F: CCC ACC GTG TTC TTC GAC A, R: CCA GTG CTC AGA GCT CGA AA. Cyclophilin was used as the endogenous housekeeping gene as it is not altered in the R6/1 mouse line at this age (Zajac, unpublished data). Real-time PCR conditions were 50°C—2 min, 95°C—10 min, followed by 40 cycles of 95°C—15 s and 60°C— 1 min. All reactions were performed on five-eight individual subjects per group, each in triplicate. Melt curve analyses were performed to ensure that only one reaction product was obtained. A total of 23 animals (12 males and 11 females) were used for gene expression analyses (n = 5–6 mice per group).

### Whole tissue measurements of 5-HT and its metabolite

Mice naïve to behavioral testing were killed for brain dissection. Tissue levels of endogenous 5-HT and its metabolite 5-hydroxyindolacetic acid (5-HIAA) were determined as previously published [39]. Dissected brain structures were homogenized in 5–10 volumes (v/w) of ice-cold 0.1 M HClO_4_ containing 1.34 mM disodium EDTA and 0.05% Na_2_S_2_O_5_. Homogenates were centrifuged at 30,000 g for 20 min at 4°C. After neutralization with 2 M KH_2_PO_4_/K_2_HPO_4_, pH 7.4, containing 0.01 mg per mL ascorbate oxidase (Boehringer Mannheim, Meylan, France), supernatants were further centrifuged at 30,000 g for 20 min. Aliquots (10 µL) of clear supernatants were injected into a high performance liquid chromatography (HPLC) column (Ultrasphere IP, Beckman, Gagny, France; 25×0.46 cm, C18 reversed phase, particle size 5 µm) protected with a Brownlee pre-column (3 cm, 5 µm). The mobile phase for the elution (at a flow rate of 1 mL per min) consisted of (in mM): KH_2_PO_4_, 70; triethylamine, 3.1; disodium EDTA, 0.1; octane sulphonate, 1.05; methanol, 16%, adjusted to pH 3.02 with solide citric acid. The electrochemical detection system (ESA 5011, Bedford, MA, USA) comprises an analytical cell with dual coulometric monitoring electrodes (+50 and +350 mV). The generated signals were integrated by a computing integrator (Millenium 32, Waters, Saint Quentin Fallavier, France). A total of 27 animals (14 males and 13 females) were used for HPLC analyses (n = 6–8 mice per group).

### Statistical Analysis

Analyses of variance (ANOVAs) were used to examine main effects and/or interactions. A 2-way ANOVA was used to analyze the effects of sex and genotype on saccharin preference, NSFT as well as measurement of gene expression (qPCR) and serotonin levels (HPLC). For the experiments involving pharmacological treatments, the effects of sex, genotype and treatment(s) were analyzed by a 3-way ANOVA. To determine specific group differences in case of significant main effects (or interaction), the ANOVAs were followed by Fisher's LSD or Bonferroni post-hoc tests. Since the latency data failed to pass the normality test (Shapiro-Wilk), group differences in the NSFT were analyzed by non-parametric tests (i.e. Kruskal Wallis test followed by a Mann-Whitney test). In all cases, the significance level was set at p<0.05. Statistical analyses were performed using SPSS statistics 17.0 and Prism 5.
